# Mixture effects of trace element levels on cardiovascular diseases and type 2 diabetes risk in adults using G-computation analysis

**DOI:** 10.1038/s41598-024-56468-6

**Published:** 2024-03-08

**Authors:** Borhan Mansouri, Ayoob Rezaei, Kiomars Sharafi, Nammamali Azadi, Meghdad Pirsaheb, Maryam Rezaei, Samaneh Nakhaee

**Affiliations:** 1 Substance Abuse Prevention Research Center, Health Institute, Kermanshah University of Medical Sciences, Kermanshah, Iran; 2 Research Center for Environmental Determinants of Health (RCEDH), Research Institute for Health, Kermanshah University of Medical Sciences, Kermanshah, Iran; 3 Biostatistics Department, School of Public Health, Iran University of Medical Sciences, Tehran, Iran; 4 Medical Toxicology and Drug Abuse Research Center (MTDRC), Birjand University of Medical Sciences, Birjand, Iran

**Keywords:** Non-communicable diseases, Heavy metals, Essential elements, G-computation, Mixed exposure, Environmental sciences, Cardiology, Diseases, Endocrinology, Health care, Medical research, Risk factors, Chemistry

## Abstract

There is an increasing concern about the health effects of exposure to a mixture of pollutants. This study aimed to evaluate the associations between serum levels of heavy/essential metals ([Arsenic (As), Cadmium (Cd), Mercury (Hg), Lead (Pb), Nickel (Ni), Chromium (Cr), Copper (Cu), Iron (Fe), and Zinc (Zn)]) and the risk of developing cardiovascular diseases (CVDs) and type 2 diabetes mellitus (T2D). Data were collected from 450 participants (150 with CVDs, 150 with T2D, and 150 healthy subjects) randomly selected from the Ravansar Non-Communicable Disease (RaNCD) cohort in Western Iran, covering the years 2018–2023. Trace element levels in the serum samples were assayed using ICP-MS. Logistic regression was performed to estimate the adjusted risk of exposure to single and multi-metals and CVD/T2D. Odds ratios were adjusted for age, sex, education, residential areas, hypertension, and BMI. The mixture effect of exposure to multi-metals and CVD/T2D was obtained using Quantile G-computation (QGC). In the logistic regression model, chromium, nickel, and zinc levels were associated with CVD, and significant trends were observed for these chemical quartiles (*P* < 0.001). Arsenic, chromium, and copper levels were also associated with T2D. The weight quartile sum (WQS) index was significantly associated with both CVD (*OR* 4.17, 95% CI 2.16–7.69) and T2D (*OR* 11.96, 95% CI 5.65–18.26). Cd, Pb, and Ni were the most heavily weighed chemicals in these models.The Cd had the highest weight among the metals in the CVD model (weighted at 0.78), followed by Hg weighted at 0.197. For T2D, the serum Pb (weighted at 0.32), Ni (weighted at 0.19), Cr (weighted at 0.17), and Cd (weighted at 0.14) were the most weighted in the G-computation model. The results showed the significant role of toxic and essential elements in CVDs and T2D risk. This association may be driven primarily by cadmium and mercury for CVDs and Pb, Ni, Cr, and Cd for T2D, respectively. Prospective studies with higher sample sizes are necessary to confirm or refute our preliminary results as well as to determine other important elements.

## Introduction

Non-communicable diseases (NCDs), also called chronic illnesses, are health conditions characterized by prolonged duration and gradual advancement. The majority of NCDs are not caused by infections and are influenced by various factors such as genetics, physiology, behavior, and the environment^1^. They are the primary cause of death globally, accounting for 74% of annual deaths^2^. The most fatal NCDs, which result in the highest number of deaths, include cardiovascular diseases (CVDs), cancer, respiratory diseases, and diabetes^2^. The CVDs and type 2 diabetes mellitus (T2D) have become a significant threat to the population's health^3^. Non-traditional risk factors such as environmental pollutants have been proposed to be linked with an increased risk of CVDs and T2D development^3–6^. Among multiple pollutants, heavy metals (HMs) have attracted excessive attention and have been widely studied due to their high toxicity, longevity, ubiquity, and adverse effects on human health^4,7^. Current evidence has reported an association between single exposure to heavy metals such as cadmium, mercury, lead, and arsenic, with an increased risk of CVDs and T2D^3^.

These toxic heavy metals are ubiquitous environmental pollutants that are not being decomposed, and they are transferred through the food chain, causing health problems for humans and other living beings^8,9^. Heavy metals are capable of causing lipid peroxidation, varying degrees of DNA damage, and protein modification. They may play a role in atherosclerosis, glucose metabolism abnormality, and beta-cell dysfunction^3,10,11^. Along with heavy metals, the role of essential trace elements (ETs) in human health should also be considered. Current evidence indicates that optimal concentrations of essential elements are important for growth, metabolism, and neurological and immunological functions, but excessive or insufficient exposure to ETs can also cause or aggravate a variety of diseases^12,13^. Also, there are growing discussions about the interactions between essential and toxic elements in the human body^14^. In the real world, human beings tend to be exposed to various metals throughout their lives. Additionally, different metals may have synergistic and/or antagonistic interactive effects on health^4,15,16^. Recently, attention has been diverted to the importance of simultaneous exposure to multiple toxicants in epidemiological investigations^12^. Few studies have focused on the mixed effects of heavy metals and essential elements on CVDs and T2D risk with conflicting results.

To enhance our understanding of the detrimental effects of exposure to various toxic and essential elements, researchers have developed several new statistical models in the field of environmental epidemiology^17^. Quantile G-computation (QGC) is among the commonly used statistical models for dealing with mixture exposure situations^17^. Considering the prevalence and importance of non-communicable diseases such as T2D and CVDs, and the limited number of studies with controversial results exploring the effect of the metal mixture on these common disorders, this study aimed to figure out the effects of mixed heavy metal/essential elements exposures on CVDs and T2D risk using the G-computation model to identify the most influential metals in the mixture.

## Materials and methods

### Study population

In the cross-sectional study, baseline data from the Ravansar Non-Communicable Disease (RaNCD) cohort in the Kurdish population of Western Iran were utilized. The RaNCD cohort study is part of the PERSIAN (Prospective Epidemiological Research Studies in IrAN) Cohort. This study was approved by the Research and Ethics Committee of Kermanshah University of Medical Sciences (IR.KUMS.REC.1401.488). All methods were performed in accordance with the relevant guidelines and regulations. Written informed consent was obtained from all participants. The subjects, aged 35 to 65 years, were divided into three groups: CVDs (n = 150), T2D (n = 150), and the control group (n = 150). The RaNCD cohort is part of the prospective epidemiological research studies in Iran (the PERSIAN Cohort Study), where 10,000 adults were selected for this cohort. More details about the RaNCD cohort have been published in previous papers^18,19^.

Inclusion criteria included people with CVDs and T2D, and people with at least two years of residence in the region. The inclusion criteria for the case group included patients with CVDs and T2D diagnosed by a specialist physician registered in the RaNCD cohort. On the other hand, the control subjects were also matched with the case subjects and were tried to be similar to the case group in terms of location and lifestyle, and the only difference was that they did not have CVDs and T2D. Patients were excluded from the study if they reported active disease before the enrollment, severe hepatic or renal dysfunction, congenital heart disease, family hereditary hyperlipidemia, malignant tumor and immune diseases, occupational exposure to heavy metals, non-native individuals, and those living in the region for less than two years. CVDs and T2D were diagnosed using clinical tests and expert examination in the RaNCD cohort.

### Elemental analysis

To evaluate the trace element concentrations in the samples, one mL of blood serum was mixed with four mL of nitric acid (HNO_3_, 65%, Suprapore, Merck, Germany) and placed in the laboratory environment for one day and night to digest slowly. On the next day, two mL of perchloric acid (HClO4, 70%, Merck, Germany) was added to the samples and heated in a Bain-Marie (TW12, Julabo GmbH, Germany) to a temperature of 98 °C for six hours. The samples were allowed to cool down and then filtered. The resultant solutions were diluted up to 25 ml with ultrapure deionized water (18.2 MΩ-cm at 25 °C, Fistreem, WSC044, UK). The levels of arsenic (As), cadmium (Cd), chromium (Cr), copper (Cu), iron (Fe), mercury (Hg), nickel (Ni), lead (Pb), and zinc (Zn) were determined using Inductively Coupled Plasma Mass Spectrometry (ICP-MS, Agilent 7900, Santa Clara, CA, USA). The certified reference material (CRM) with the same matrix and elements tested was not found on the market. Therefore, the CRM BCR-185R and spikes were used (recoveries for all the elements fell between 92 and 105%, N = 4). The final results were expressed as µg/L of serum.

### Statistical analysis

The characteristics of participants were reported using mean ± standard deviation (SD) for numerical variables or frequencies and proportions for categorical variables. The chi-squared test was used to compare frequencies, and the Kruskal–Wallis test was used to compare concentration levels between the three groups. To deal with the skewed distribution of metal levels, all element/metal concentration levels were log-transformed and standardized to have mean zero and variance one. Concentration levels were described as the geometric mean (GM) and 95% confidence interval (CI). A logistic regression model was used to assess the relationship between each element and CVD/T2D by comparing the second, third, and fourth quartiles of concentration levels to the first quartile. Additionally, the Pearson correlation coefficients between standardized levels of the elements were analyzed. Furthermore, Quantile G-Computation was used to investigate the mixture effect of all elements/metals. The mixture effects were estimated using the qgcomp.no boot function from the qgcomp package in R. A p-value of less than 0.05 was considered significant.Weighted quantile sum (WQS) regression, developed specifically for the context of environmental mixtures analysis, is a widely used technique to estimate the individual and cumulative effect of multi-pollutant exposure on the outcome. A WQS regression model takes the following form:

$$g\left(\mu \right)={\beta }_{0}+{\beta }_{1}\left(\sum_{i=1}^{c}{w}_{i}{q}_{i}\right)+{z}{\prime}\varphi$$

where ${w}_{i}$ is an unknown weight associated to each metal exposure reflecting its relative importance, ${q}_{i}$ is the metal concentrations transformed into quartiles, ${\beta }_{1}$ is the coefficient estimating joint effect of metal exposures. The z′ represents the vector of covariates and $\varphi$ their coefficients. One drawback of the WQS regression is its constrained on the direction of the exposure effects. It assumes that the metal exposures affect the outcome in one-direction only. That is all exposures have a positive (or negative) effect on CVD/T2D. To overcome this shortage of the WQS regression model, several extensions to this model has been proposed. Quantile g-computation is a generalized version of WQS that uses g-computation to estimate the weights and the mixture effect of heavy metals exposures on CVD/T2D risk. Both WQS and quantile g-computation score the heavy metal concentrations into quantiles and use generalized linear models to estimate the exposure effects with reference to the lowest quantile.

### Ethics approval and consent to participate

This study was approved by the Research and Ethics Committee of Kermanshah University of Medical Sciences (IR.KUMS.REC.1401.488). Written informed consent was obtained from all participants.

## Results

### Study characteristics

In the study, the population comprised 150 diabetic patients, 150 cardiovascular patients, and 150 healthy subjects without signs of diabetic or cardiovascular diseases. Among the cardiovascular disease patients, 60% were uneducated and 30.7% had less than a 12th-grade education. They were mostly of higher age levels (mean age 52.8), predominantly male (54%), and resided in rural areas (55.3%). 40% of the cardiovascular disease cases also suffered from hypertension. The diabetic subjects were younger than the cardiovascular disease cases (mean age 48.16), mostly female (55.3%), and uneducated (54.67%), and resided in urban areas (64.7%). The groups were comparable in terms of gender distribution. Overall, the majority of subjects had normal blood pressure, with 73% having no hypertension. As expected, the Type 2 Diabetes cases were obese with an average BMI of 30.91. Demographic information stratified by the study groups is provided in Table 1.

**Table 1:** Characteristics of the study population in a case–control study of Ravansar, Persian Cohort Study, 2018–2023.

Variables	Controls (n = 150)	CVD cases (n = 150)	Diabetic cases (n = 150)	*P* values
	Mean ± SD or n (%)	Mean ± SD or n (%)	Mean ± SD or n (%)	
Age (years)	45.78 ± 7.95	52.77 ± 7.89	48.16 ± 7.42	< 0.001
Sex (%)				
Male	76 (50.67)	81 (54)	67 (44.67)	0.261
Female	74 (49.33)	69 (46)	83 (55.33)	
Education levels (%)				
Illiterate	66 (44)	98 (65.33)	82 (54.67)	< 0.001
Less than 12th grade	64 (42.67)	46 (30.67)	47 (31.33)	
High School Grade or above	20 (13.33)	6 (4)	21 (14)	
BMI (kg/m2)	27.09 ± 4.47	27.60 ± 4.58	30.92 ± 4.52	< 0.001
Residential areas (%)				
Rural	57 (38)	83 (55.33)	53 (35.33)	< 0.001
Urban	93 (62)	67 (44.67)	97 (64.67)	
Hypertension				
No	132 (88)	90 (60)	108 (72)	< 0.001
Yes	18 (12)	60 (40)	42 (28)	

**Figure 1:**
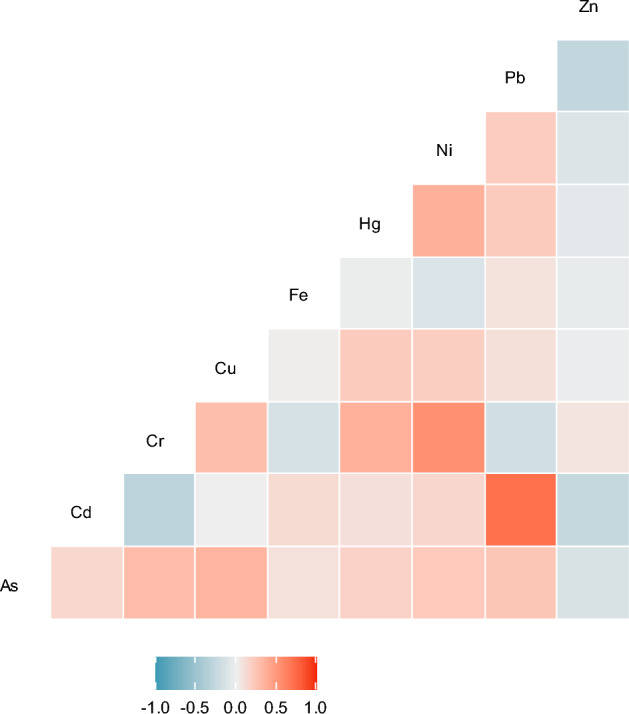
Pairwise Spearman correlations among serum concentrations of metals or elements (n = 450), Ravansar, Persian cohort, Iran. As, arsenic; Cd, cadmium; Cr, chromium; Cu, copper; Fe, iron; Hg, mercury; Ni, nickel; Pb, lead; Zn, zinc.

The pairwise associations between the serum levels of metals/elements were investigated using Spearman correlation coefficients (r). Figure 1 displays the heatmap-colored correlation matrix of metal/element levels (r ranging from − 0.301 to 0.704). The correlations were mostly positive and ranged from weak to moderate. There was a strong positive correlation between Cd and Pb (r = 0.704).

Table 2 represents the mean and quartiles of each trace element level, along with a comparison of concentration levels among the three studied groups using Kruskal–Wallis test. It appears that diabetic subjects had higher serum concentration levels of As, Cr, Cu, Hg, and Ni compared to the cardiovascular disease and control groups (*P* < 0.001). Cardiovascular disease cases were diagnosed with higher levels of Fe (*P* = 0.01) and Pb (*P* < 0.001) compared to Type 2 Diabetes and control groups. In contrast to the case groups, control subjects had higher serum levels of Zn (*P* < 0.001).

**Table 2:** Metal/essential element distribution (µg/L or mg/L) in the case–control study of the effect of trace elements on CVD/diabetic disorder, Ravansar, Persian cohort, Iran.

Metal/element	Group	Mean (95% CI)	25%	50%	75%	*P* value
As	Control	1.88 (1.41–1.50)	0.20	3.8	8.07	< 0.001
	CVD	4.39 (3.84–5.03)	2.57	5.01	7.52	
	Diabetic	9.76 (8.74–10.91)	7.05	11.10	15.0	
Cd	Control	1.38 (0.13–0.15)	0.1	0.1	0.20	< 0.001
	CVD	2.53 (2.23–2.88)	2.7	2.83	3.30	
	Diabetic	0.69 (0.57–0.83)	0.4	0.90	1.47	
Cr	Control	11.3 (10.05–12.7)	6.8	9.8	16.25	< 0.001
	CVD	3.07 (2.67–3.52)	1.64	2.31	5.26	
	Diabetic	39.01 (35.4–43.03)	27.3	41.3	61.1	
Cu	Control	58.2 (54.5–62.2)	42.2	63	81.8	< 0.001
	CVD	54.5 (50.8–58.5)	41.5	55.6	75.8	
	Diabetic	77.9 (74.0–82.0)	66.3	84.1	101.3	
Fe	Control	483.2 (442.1–528.2)	353.3	474.7	731.5	0.010
	CVD	537.9 (512.2–565.0)	429.9	531.8	628.0	
	Diabetic	466.0 (436.2–497.9)	350.3	458.1	640.3	
Hg	Control	0.98 (0.906–1.06)	0.92	1.20	1.30	< 0.001
	CVD	1.23 (1.09–1.40)	0.67	1.01	2.26	
	Diabetic	1.81 (1.67–1.96)	1.12	1.85	2.90	
Ni	Control	6.51 (5.62–7.53)	3.22	5.30	17.3	< 0.001
	CVD	10.29 (9.04–11.7)	7.36	11.7	17.8	
	Diabetic	27.6 (25.9–29.4)	20.5	26.3	36.6	
Pb	Control	1.51 (1.32–1.72)	0.80	1.60	2.70	< 0.001
	CVD	13.29 (12.9–13.6)	11.56	12.7	14.8	
	Diabetic	8.86 (8.23–9.54)	6.35	8.95	12.2	
Zn	Control	679.6 (626.1–737.8)	458.6	838.7	1032.1	< 0.001
	CVD	480.8 (444.3–520.4)	314.3	514.8	736.2	
	Diabetic	519.9 (466.4–579.6)	425.4	669.9	789.6	

As, arsenic; Cd, cadmium; Cr, chromium; Cu, copper; Fe, Iron; Hg, mercury; Ni, nickel; Pb, lead; Zn, zinc. CVD, cardiovascular disease.

### The relationship between trace elements and CVD using the regression model

The effect of each single element on developing CVD and T2D is presented in Tables 3 and 4, respectively. Results were obtained by adjusting for sex, age, residence area, education, BMI, and hypertension covariates under a multiple logistic regression model. The results suggest a link between the serum levels of Cr (P for trend = 0.010), Ni (*P* < 0.001), and Zn (*P* < 0.001) with cardiovascular disease. There is also a significant association between As levels and developing CVD (*p* for trend 0.010). For As and Cr, the ORs are significant at the 2nd, 3rd and 4th quartiles when compared to the first quartile. The levels of Fe and Hg show a significant association with CVD on the second and third quantiles, but the general trend isn’t significant (*p* for trend 0.151 and 0.617). The levels of Ni show a significant association with CVD on the third and fourth quantiles. The ORs for Cu are not significant in any quartile. Zn is only significant at quantile 4, but its trend is significant (*p* trend < 0.001). Due to issues with logistic model convergence, the effect of Cd and Pb wasn’t evaluated.

**Table 3:** Association between single serum metal/element concentration and cardiovascular disease, Ravansar, Iran**.** Adjusted for gender, sex, residence area, hypertension, education levels, and BMI.

Metal	Model 1								Model 2	
	Q1	Q2		Q3		Q4				
		OR (95% CI)	*P* value	OR (95% CI)	*P* value	OR (95% CI)	*P* value	P-trend	OR (95% CI)	*P* value
As	Ref	9.8 (4.2–24.2)	< 0.001	4.9 (2.2–11.4)	< 0.001	4.5 (2.1–10.6)	< 0.001	0.010	1.05 (1.003–1.11)	0.048
Cr	Ref	0.01(0.001–0.09)	< 0.001	0.001 (0.00007–0.009)	< 0.001	0.001 (0.0001–0.008)	< 0.001	< 0.001	0.76 (0.69–0.81)	< 0.001
Cu	Ref	1.09 (0.52–2.31)	0.816	0.76 (0.36–1.59)	0.474	0.52 (0.24–1.09)	0.088	0.067	0.98 (0.97–0.99)	0.042
Fe	Ref	6.01 (2.86–14.1)	< 0.001	9.07 (3.97–21.9)	< 0.001	1.70 (0.75–3.93)	0.205	0.151	1.01 (0.99–1.02)	0.339
Hg	Ref	0.40 (0.18–0.86)	0.020	0.14 (0.06–0.33)	< 0.001	1.92 (084–4.51)	0.125	0.617	1.80 (1.03–3.37)	0.001
Ni	Ref	1.25 (0.57–2.77)	0.566	18.2 (7.4–48.8)	< 0.001	3.47 (1.57–7.96)	0.002	< 0.001	1.06 (1.02–1.09)	0.001
Zn	Ref	1.75 (0.79–3.93)	0.163	0.84 (0.38–1.84)	0.656	0.04 (0.013–0.11)	< 0.001	< 0.001	0.997 (0.995–0.998)	< 0.001

*Model 1: Concentration levels were quantized and comparisons were made between the second, third, and fourth quantiles to the first quantile. Model 2: Scaled concentration levels were used. As, arsenic; Cd, cadmium; Cr, chromium; Cu, copper; Fe, iron; Hg, mercury; Ni, nickel; Pb, lead; Zn, zinc.

**Table 4:** Association between single serum metal/element concentration levels and T2D, Ravansar, Iran**.** Adjusted for gender, sex, residence area, hypertension, education levels, and BMI.

Metal	Model 1								Model 2	
	Q1	Q2		Q3		Q4				
		OR (95% CI)	*P* value	OR (95% CI)	*P* value	OR (95% CI)	*P* value	P trend	OR (95% CI)	*P* value
As	Ref	8.7 (3.4–24.3)	< 0.001	20.8 (8.1–59.2)	< 0.001	39.9 (15.1–118.9)	< 0.001	< 0.001	1.22 (1.16–1.30)	< 0.001
Cr	Ref	3.14 (1.23–8.29)	0.002	1.89 (4.5–13.6)	< 0.001	4.21 (2.26–6.12)	< 0.001	< 0.001	1.12 (1.09–1.15)	< 0.001
Cu	Ref	3.13 (1.43–7.10)	0.005	3.35 (1.52–7.65)	0.003	10.5 (4.58–25.7)	< 0.001	< 0.001	1.04 (1.02–1.05)	< 0.001
Fe	Ref	1.17 (0.56–2.46)	0.663	1.16 (0.56–2.41)	0.668	0.91 (0.43–1.89)	0.792	0.469	0.99 (0.98–1.001)	0.332
Hg	Ref	0.64 (0.29–1.3)	0.244	1.32 (0.54–3.17)	0.534	2.02 (0.89–3.15)	0.632	0.231	7.1 (2.32–14.6)	< 0.001
Zn	Ref	1.25 (0.59–2.65)	0.553	1.32 (0.63–2.78)	0.455	0.26 (0.17–0.56)	0.001	0.003	0.998 (0.997–0.999)	0.0002

*Model 1: Concentration levels were quantized and comparisons were made between the second, third, and fourth quantiles to the first quantile. Model 2: Scaled concentration levels were used.

Apart from As and Cu, which are marginally significant, significant associations between serum levels of Cr, Hg, Ni, and Zn with CVD are found when considering the element levels as continuous variables (Table 3).

Investigating the association between the levels of metals/elements and T2D in Table 4 shows a significant effect for As, Cr, Cu, and Hg (*p* trend < 0.001). Zn is only significant at quantile 4, but its trend is significant (*p* trend 0.003). For As, Cr, and Cu, the ORs are significant at the 2nd, 3rd and 4th quartiles when compared to the first quartile. The ORs for Fe and Hg are not significant in any quartile. The logistic model didn’t converge for Ni, Cd, and Pb; therefore, they are excluded from Table 4.

### The mixture effect of elements:

The mixture effect (obtained using G-computation indices) is significantly associated with both CVD and diabetes development (Table 5). After adjusting for sex, age, residence area, education, BMI, and hypertension, it is found that a quartile increase in the qgcomp index is significantly associated with CVD (OR 4.17, 95% CI 2.16–7.69) and T2D (OR 11.96, 95% CI 5.65–18.26). The estimated metal/element weights for the CVD and T2D qgcomp index are presented in Tables 6 and 7, respectively. The Cd is the metal with the highest weight in the CVD model (weighted at 0.781), followed by Hg weighted at 0.197. Cu element is given the lightest weight. After adjusting for all covariates (Tables 5 and 7), a quartile increase in the qgcomp index is associated with an 11.96 increase in developing diabetes (95% CI 5.65–18.26). Table 7 shows the weights of each element with serum Pb level, Ni, Cr, and Cd as the most weighted. Fe has the lightest weight.

**Table 5:** Association between WQS regression index and CVDs/DMs, Ravansar, Iran. Adjustment for CVDs/DMs.

Models	OR (95% CI)	*P* value
Model 1: CVD vs Control	4.17 (2.16–7.69)	0.019
Model 2: Diabetes vs Control	11.96 (5.65–18.26)	< 0.001

**Table 6:** G-computation index weights estimated for CVDs, Ravansar, Iran.

Exposure element	The scaled effect size for CVDs	
	Positive direction	Negative direction
Cd	0.7810	
Hg	0.1977	
Fe	0.0213	
Cr		0.6823
Zn		0.1566
As		0.1156
Cu		0.0455

**Table 7:** G-computation index weights estimated for diabetics in Ravansar, Iran.

Exposure element	The scaled effect size for diabetic patients	
	Positive direction	Negative direction
Pb	0.3244	
Ni	0.1955	
Cr	0.1717	
Cd	0.1442	
As	0.0643	
Hg	0.0513	
Cu	0.0354	
Zn	0.0131	
Fe		1

## Discussion

### The mixture and single effects of trace elements on CVDs

In this study, the G-computation model was implemented to identify mixed heavy metal/essential element exposures that contribute significantly to cardiovascular diseases and T2D. CVD incidence was positively correlated with serum concentrations of Cd and Hg as the most weighted elements, and inverse associations were observed for Cr concentrations. The results of previous literature are contradictory in this field. Some reports have indicated that exposure to metal mixtures is correlated with cardiovascular diseases^20–22^. Guo et al.^4^ studied the mixed effects of toxic heavy metals on CVD to find the most predominant metals in the mixture. Five statistical models were applied, and it was found that cadmium, cobalt, tungsten, and antimony showed the strongest positive relationships, whereas lead, thallium, barium, and molybdenum had the most negative correlations with CVD^4^. In a prospective investigation using a Bayesian kernel machine regression (BKMR), it has been found that co-exposure to nine heavy metals was associated with CVD incidence, and cadmium and antimony were the main elements of the mixture^21^. Furthermore, exposure to multiple heavy metals simultaneously was reported to be correlated with impairment of cardiac autonomic functions. The authors fitted linear mixed-effects models and reported that mercury (Hg) was probably the main influential element in metallic components^23^. Liu et al.^17^ evaluated the relationship of single and multiple metals with the biomarker of early cardiovascular damage, Mean Platelet Volume (MPV), using a combination of five statistical models, including Quantile G-computation, general linear models, BKMR, adaptive elastic network regression (AENR), and weight quartile sum (WQS) regression, among 3396 adults. In conclusion, single metals may be negatively correlated with early cardiovascular damage (arsenic, antimony, and iron) and positively correlated with early cardiovascular damage (thallium, aluminum, and tungsten). The association of mixed metals with MPV in the simultaneous exposure to twenty-three elements in the G-computation model was insignificant^17^.

Park et al.^20^ extended the environmental risk score (ERS) by considering pollutant-pollutant interactions using machine learning (ML) models. They assessed twenty metal biomarkers in urine or whole blood from 6 courses of the National Health and Nutrition Examination Survey. ERS established by the model containing several metals as a mixture demonstrated a positive correlation with cardiovascular endpoints (systolic and diastolic blood pressure and hypertension)^20^. Evidence from some meta-analyses generally highlights that elevated levels of cadmium^24–26^, mercury^27^, arsenic^28^, and lead^29^ were associated with a higher risk of cardiovascular events.

Another meta-analysis study found that cadmium, lead, arsenic, and copper exposure was associated with an elevated risk of CVDs and coronary heart disease. However, Hg was not associated with cardiovascular disease risk^29^. Results evaluating metal exposure and cardiovascular diseases have been less consistent, which can be due to assessing heavy metals/trace elements in different methods and different sample sizes, as well as not taking into account adjusting the potential effect of confounding variables.

### The mixture and single effects of trace elements onT2D:

Our results revealed that the most weighted elements positively associated with the risk of developing T2D were Pb, Ni, Cr, and Cd. In a Prospective study, the incidence of diabetes was followed up for fifteen years. Six different machine learning algorithms were used to investigate whether the combination of pollutants could predict the incidence of diabetes. They found that machine learning helped find combinations of pollutants important for predicting diabetes. Nickel was a heavy metal most closely associated with diabetes incidence over 5 years (OR 1.44, 95% CI 1.05–1.95, *P* = 0.022)^30^. Another study on the effects of 8 urinary metals on T2D progression was evaluated using the glycemic status of 414 prediabetic and normoglycemic adults. The QGC model was used to determine the mixture effects. Their data highlighted that arsenic, copper, and molybdenum were associated with glucose homeostasis alterations and higher levels of the eight metal mixtures were related to lower plasma insulin levels, HOMA-IR, and HOMA-β as well as higher HOMA-S^31^.

Some researchers assessed the effects of metal mixture on gestational diabetes mellitus (GDM). For example, Wang et al. (2020) used the BKMR model to recognize the joint effects and the possible interactions between metals. Mixed exposure of six studied metals (Ni, Sb, As, Cd, Co, and V) was positively associated with increased risk of GDM, while Ni and antimony showed more important effects than the other 4 elements in the mixture^32^. In another study, the data of 78,964 pregnant women were used. QGC, logistic regression analysis, and distributed lag nonlinear model (DLNM) were conducted to investigate the relationship between the blood levels of different metals and GDM risk. In the logistic regression model, maternal blood mercury was related to an elevated risk of GDM. In the QGC model, although the mixture of elements was not associated with an increased GDM risk, mercury (52.6%) may be the main factor^33^. Another data determined that manganese may be the main risk factor for GDM and nickel the main protective element against the risk of GDM in the Quantile G-computation model^34^.

Some studies have focused on single exposure effects of heavy metals/trace elements on diabetes with inconsistent results^3,12,35^. Considering the different types of elements included in metal mixtures across previous studies and the heterogeneity of bio-samples (e.g., serum, plasma, or urine), the deleterious effect of exposure to metal mixture on CVD and T2D needs to be explored. Previous investigations typically included individuals with different characteristics and lifestyle risk factors, such as dietary patterns and exposure to heavy metals from different sources, which may lead to different results. Some studies had relatively small sample sizes, which may not provide statistical power for detecting reliable associations. The biological roles or possible mechanisms of different heavy metals/essential elements with CVDs or T2D need to be further investigated.

### Trends observed in the chemical quartiles:

Based on the results of the present study, some of the metals (As and Cr) in all quartiles (both high and low values) were associated with the risk of heart disease and diabetes, while some others only increased or decreased the risk in the high or middle quartiles. The results of a study indicate a non-linear relationship between plasma arsenic and the development of coronary heart disease (CHD). There was a significant positive association for the highest quartile of exposure (μ > 3.49 μg/L vs. μ < 1.28 μg/L), while the second and third quartiles showed ORs close to the null. The spline regression model also showed a positive association with concentrations μ > 3.7 μg/L^36^. Similar patterns were observed in the Strong Heart Study^37^ and the HEALS study^38^.

Hansen et al.^39^ found in a nested case–control study that the prevalence of previously undiagnosed type 2 diabetes increased across quartiles for cadmium, chromium, iron, nickel, silver, and zinc, and decreased with increasing quartiles of bromine (P trend < 0.05). After correcting for multiple testing, associations for chromium remained significant (Q trend < 0.05)^39^. Further research is necessary to determine the threshold and reference dose of trace metal exposure and its impact on cardiovascular disease (CVD) and type 2 diabetes (T2D) risk. Our data suggests a positive association between plasma arsenic and chromium with the incidence of CVD and T2D in adults, even at relatively low to moderate exposure levels.

### Potential mechanisms

There are general mechanisms that are applicable to all toxic metals, as well as specific mechanisms that are unique to each individual metal. These mechanisms primarily revolve around oxidative stress^40,41^. Many metals have the ability to form covalent bonds with sulfhydryl groups of proteins due to their electron-sharing properties^40,42^. The binding to glutathione leads to depletion of its levels, resulting in an increase in the intracellular concentration of reactive oxygen species (ROS). The consequences of this process include the promotion of lipid peroxidation, damage to cell membranes, DNA damage, oxidation of amino acids in proteins leading to changes in their structure and function, and inactivation of enzymes^40^. Other potential biological mechanisms for the effects of heavy metals on the cardiovascular system include altered regulation of endocrine and endothelial vascular functions, as well as epigenetic pathways^41^. For instance, the effects of cadmium on the cardiovascular system may involve oxidative stress, inflammation, endothelial dysfunction, up-regulation of adhesion molecules, enhanced lipid synthesis, prostanoid dysbalance, and altered glycosaminoglycan synthesis^24^. Lead, along with other metals, can deactivate paraoxonase and consequently promote LDL oxidation and the development of atherosclerosis^40^.

The main mechanisms of lead toxicity have been extensively studied, including its ability to substitute other bivalent elements such as Ca^2+^, Fe^2+^, and Zn^2+^, as well as its role in causing oxidative stress^42^. Lead competes with zinc and binds to sulfhydryl groups of delta-aminolevulinic acid dehydratase, preventing the binding of this enzyme to aminolevulinic acid and generating ROS^40,42^. The oxidative attack on cellular components by ROS is implicated in the development of several human diseases, including diabetes^43^.

### Strengths and limitations

Considering several essential trace elements and heavy metals in serum specimens with validated methods, and evaluating their association with two common outcomes is a major strength of the current study. Additionally, this study considered the effects of multiple metals to investigate their interactions, resulting in more reliable estimates. However, this study has limitations. For instance, we lacked detailed information about the participants' dietary patterns and the measurements of essential elements in their food. We cannot rule out the possible effects of changes in lifestyle, metabolism, or medication use after developing diabetes/CVDs on the absorption or excretion of some trace elements/metals. Therefore, the adjustment for many potential confounders, including lifestyle and sociodemographic variables, was not performed. Also, a prospective study with a follow-up period to establish more reliable causality was not conducted.These limitations underscore the need for larger prospective studies with more extensive data collection efforts to better elucidate the complex interplay between trace elements/metals and health outcomes. Additionally, a prospective study with a longer follow-up period would be imperative to establish more robust causality and to capture potential changes in trace element levels over time.

## Conclusion

Overall, our study illustrates several associations between HMs and TEs with CVDs and T2D. It provides more evidence for the probable significance of toxic and essential elements in these common non-communicable disorders. The heavy metals that most contribute to the increased risk of CVDs and T2D are Cd, Hg, Cr, Pb, and Ni. The incidence of CVD was positively correlated with serum concentrations of Cd and Hg as the most heavily weighted elements in G-computation model, and inverse associations were observed for Cr concentrations. The most heavily weighted elements positively associated with the risk of developing T2D were Pb, Ni, Cr, and Cd. Prospective studies are warranted to validate our results and clarify the potential mechanisms and observed trends. In communities affected by disproportionate environmental and occupational exposure, surveillance systems should monitor metal biomarkers and diabetes/cardiovascular disease events and implement prevention programs. Since metals are associated with cardiovascular disease and type 2 diabetes, even at relatively low levels of exposure, population-wide strategies to minimize exposure can further contribute to overall non-communicable disease prevention efforts.

## Data Availability

The data used and analyzed in the current study are available from the corresponding author upon reasonable request.

## Code availability

No custom code was used**.**
